# Theranostic Terbium Radioisotopes: Challenges in Production for Clinical Application

**DOI:** 10.3389/fmed.2021.675014

**Published:** 2021-05-31

**Authors:** Nabanita Naskar, Susanta Lahiri

**Affiliations:** Chemical Sciences Duvision, Saha Institute of Nuclear Physics, Kolkata, India

**Keywords:** ^149,152,155,161^Tb, theranostic radioisotopes, light charged particle activation, heavy ion activation, spallation reaction, radiochemical separation

## Abstract

Currently, research on terbium has gained a momentum owing to its four short-lived radioisotopes, ^149^Tb, ^152^Tb, ^155^Tb, and ^161^Tb, all of which can be considered in one or another field of nuclear medicine. The members of this emerging quadruplet family have appealing nuclear characteristics and have the potential to do justice to the proposed theory of theranostics nuclear medicine, which amalgamates therapeutic and diagnostic radioisotopes together. The main challenge for *in vivo* use of these radioisotopes is to produce them in sufficient quantity. This review discusses that, at present, neither light charged particle nor the heavy ion (HI) activation are suitable for large-scale production of neutron deficient terbium nuclides. Three technological factors like (i) enrichment of stable isotopes to a considerable level, (ii) non-availability of higher energies in commercial cyclotrons, and (iii) non-availability of the isotope separation technique coupled with commercial accelerators limit the large scale production of terbium radionuclides by light charged particle activation. If in future, the technology can overcome these hurdles, then the light charged particle activation of enriched targets would produce a high amount of useful terbium radionuclides. On the other hand, to date, the spallation reaction coupled with an online isotope separator has been found suitable for such a requirement, which has been adopted by the CERN MEDICIS programme. The therapeutic ^161^Tb radionuclide can be produced in a reactor by neutron bombardment on enriched ^160^Gd target to produce ^161^Gd which subsequently decays to ^161^Tb. The radiochemical separation is mandatory even if the ISOL technique is used to obtain high radioisotopic purity of the desired radioisotope.

## Introduction

The discipline of “Nuclear Medicine” has passed through a “series of growth phases” since its inception. The present growth phase of nuclear medicine is about the fascinating progress of the discipline in the direction of theranostics (1).

$$\begin{array}{r} \text{Theranostics }=\text{ Therapeutic }+\text{ Diagnostic} \end{array}$$

The term “theranostics” was first coined by John Funkhouser in a 1998 press release, in the context of personalized treatment (2, 3). It is a holistic and tailor-made pharmacotherapy that enhances the therapeutic effects with efforts to reduce treatment toxicities. In nuclear medicine, theranostics refers to the pairing of therapeutic-diagnostic radioactive candidates chelated to a compound (carrier/vector) and targeted toward any particular clinical condition. Therapeutic radioisotopes decay by releasing a particle like α/β^−^/auger electrons, which are capable of ionization or bond-breakage, whereas diagnostic radioisotopes decay by releasing gamma rays or emit gamma rays after annihilation of β^+^, which are used for imaging purposes. Ideally, a theranostic pair in nuclear medicine should be composed of radioisotopes derived from the same element, one serving as a therapeutic agent and another aiding in diagnosis. Practically, such conjugation has to pass through stringent scrutiny before being referred to as a proper theranostic radiopharmaceuticals (RP), (RP = Radiotracer + Carrier/Vector).

While explaining such theranostic candidates, Schottelius et al. (4) have used the term “twins in spirit” for a pair which may or may not be chemically or biologically identical but the diagnostic counterpart can effectively predict the bio-distribution of the therapeutic radionuclide. A few examples would be helpful to visualize the concept of a “matched pair.” So-called matched pairs or theranostic pairs include: ^43/44^Sc–^47^Sc, ^64^Cu–^67^Cu, ^72^As–^77^As, ^83^Sr–^89^Sr, ^86^Y–^90^Y, ^124^I–^131^I, ^203^Pb–^212^Pb, etc. (5–8). All of these pairs have the combination of β^+^-β^−^. ^86^Y–^90^Y was the first matched pair being used for theranostic purposes. In this pair, ^86^Y (*T*_1/2_ = 14.7 h) provided the β^+^ (β^+^ = 31.9%, EC = 68.1%) used for imaging and ^90^Y (*T*_1/2_ = 2.7 d) is the β^−^ emitter (100%) that acted as the therapeutic part. Presently, the theranostic pair of ^68^Ga-^177^Lu has achieved great success and is in routine use for treatment of neuroendocrine tumors (NET). ^68^Ga (*T*_1/2_ = 67.6 min; 89% positron branching) is a common PET candidate and is readily available from the ^68^Ge/^68^Ga generator system. ^68^Ga-tracers, chelated with ligands like peptides, proteins, or antibodies, are in use for several diagnostic applications (9). On the other hand, the therapeutic counterpart, ^177^Lu (*T*_1/2_ = 6.7 d) is a beta-emitter. In a ^68^Ga-^177^Lu combination, ^68^Ga does the imaging along with receptor visualization and antigen expression and ^177^Lu is utilized for radiotherapy (10). For treatment of NET in patients, peptide receptor radionuclide therapy (PRRT) is usually preferred. In PRRT, peptide molecule like octreotide (somatostatin analog), covalently bound to chelators (DOTA, NOTA, etc.), enables the coordination of β^+^(^68^Ga)-β^−^(^177^Lu) candidates.

Though matched pairs hold a brighter prospect and some combinations are in the pre-clinical or trial phase, broad level administration is still a constraint and requires more experiments, practical knowledge, and easy availability of the concerned radioisotopes.

In recent years, research on various terbium radionuclides has been carried out mainly by the physics and chemistry community in and around Geneva. Terbium is referred as the “*Swiss knife*” because of its four valuable radioisotopes, ^149^Tb, ^152^Tb, ^155^Tb, and ^161^Tb. Despite their lucrative nuclear properties useful in diagnosis and therapy and their evolvement as successful theranostic pair; radionuclidic therapy with terbium radionuclides is still a challenge due to their low production cross section. Due to unavailability of these radionuclides in sufficient quantities, at the moment, only few works have been reported related to pre-clinical and clinical studies with terbium radionuclides.

After a brief introduction of these four radionuclides, this review discusses the methods of production of important terbium radionuclides by light and heavy ion induced reactions as well as a spallation reaction followed by the separation of these radionuclides from the target matrix whenever required.

## Brief Introduction to Radiotherapy

**Figure 1:**
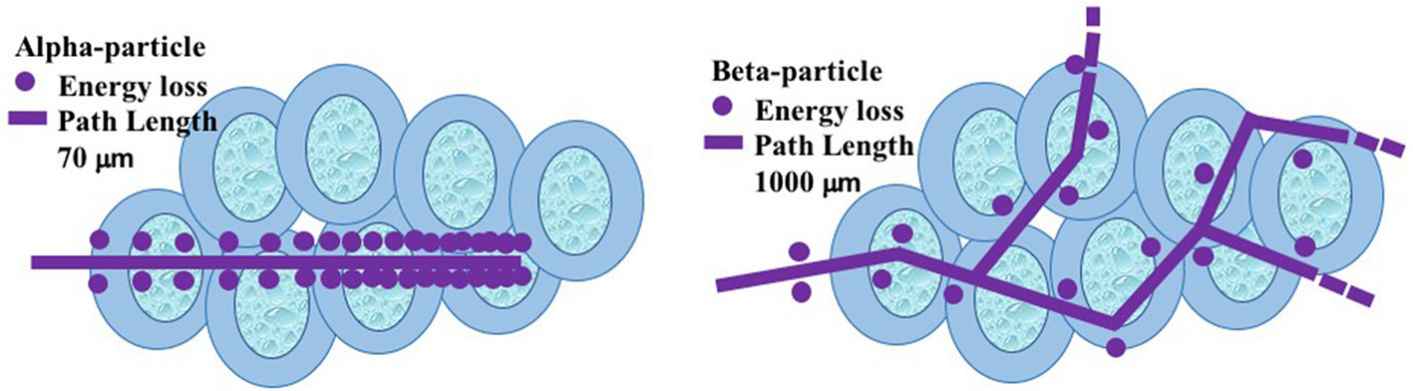
Path lengths of alpha- and beta- particles at target site.

Radiotherapy can be achieved in conjugation with α-, β-, or Auger electron emitters. The α-particles have high energy (~4–9 MeV), and high linear energy transfer (LET >20 to hundreds of keV/μm). The possibility of ionization per unit path length is very high for α-particles and therefore cytotoxicity is 5–100 times higher as compared to β-particles. Because of higher cytotoxicity and the probability of a large number of ionizations, only with few α-particle emissions, effective cell killing is achieved (11) (Figure 1). Alpha particles are generally suitable for small tumors, isolated, or micro-clustered tumors because of their short path-length (40–100 μm; ~1–3 cell diameter). The α-emitting radionuclides like ^211^At (7.21 h), ^212^Bi (60.55 min), ^213^Bi (45.6 min), ^225^Ac (10 d), ^212^Pb (10.64 h), ^223^Ra (11.43 d), and ^149^Tb (4.12 h) can essentially be used in cancer therapy.

On the other side, the β-particles have medium to high mean energy (0.5–2.3 MeV) and low LET (~0.2 keV/μm). They have a longer path-length (μm to few cm, i.e., ~5–150 cell diameter), are approximately in tissue-level range, and therefore may be suitable for large tumors or macro-clusters.

Use of Auger electrons was first proposed by Feinendegen (12). The Auger electrons are very low-energy electrons (eV-keV) having considerable LET. Their path-length is at subcellular range (few μm). If the Auger electron emitting radionuclide is internalized in the cell nucleus with the help of a suitable vector, then maximum energy deposition occurs close to the cell nucleus. The conversion or Auger electrons are generally suitable for isolated or micro-clusters (13–15). Therapy with Auger electrons is still at its nascent phase and requires much more understanding related to its bio-distribution kinetics at the subcellular level.

The alpha particles have high LET, lower path length/range and high relative biological effectiveness (RBE) (16). Alpha particles are capable of creating dense ionization tracks on DNA double strands that results in clusters of DNA damage. Such complex damage results in chromosome aberration, impairment in reproductive integrity of any cell (cell cycle arrest is shorter), etc. Such damages are more genotoxic and resistant to normal repair, resulting in high probability of cell death. Approximately 100–200 keV/μm LET is required for double strand break at the maximum rate, whereas low LET or gamma radiations result in sparsely distributed DNA breaks (17–20). It is noteworthy to mention that the beta particles and alpha particles are complementary to each other, the former is more interesting for diffuse or residual diseases, the latter one can be used in mm size clusters of cancer cells. However, to date, β^−^ emitters are more popular in therapy rather than α emitting radionuclides.

## Introduction to Terbium Radionuclides

Some of the radioisotopes of the lanthanide series exhibit suitable half-lives and distinct modes of decay schemes relevant to nuclear medicine (Table 1). The importance of radio-lanthanides in the field of nuclear medicine was elaborately reviewed in 1999 (22). In the last 20 years, the radioisotopes of two lanthanide elements, Tb and Lu, came in the forefront. ^177^Lu is now regularly being used in hospitals for *in vivo* administration to the patients for therapy. While four radioisotopes of terbium, ^149^Tb, ^152^Tb, ^155^Tb, and ^161^Tb came into the center stage of discussion and have been revealed as some of the most powerful tools for both therapy and diagnosis in the near future. This review focuses on these four radioisotopes of terbium in the following section.

**Table 1:** Some potential radio-lanthanides proposed in nuclear medicine.

Radio-lanthanides (half-life)^*^	Decay modes (branching ratio)^*^	Gamma-energy, keV (I_γ_, %)^*^	β_end point_ energy, keV (I_γ_, %)^*^	**Application**
^134^La (6.5 min)	β^+^ (63%); EC (37%)	605 (5.0), 511.0 (127.2)	2,709 (62.0)	Imaging
^140^La (1.7 d)	β^−^ (100%)	328.8 (20.3), 487.0 (45.5), 815.8 (23.3), 1596.2 (95.4)	1,239 (11.0), 1,348 (43.9), 1,677 (20.2)	Therapy
^141^Ce (32.5 d)	β^−^ (100%)	145.44 (48.2)	435 (69.7), 580 (30.3)	Tracer studies
^140^Pr (3.4 min)	β^+^ (51%); EC (49%)	511.0 (102.0)	2,366 (51.0)	Imaging
^143^Pr (13.6 d)	β^−^ (100%)	No good gamma-energy	934 (100)	Therapy
^144^Pr (17.3 min)	β^−^ (100%)	No good gamma-energy	2,997 (97.9)	Therapy
^149^Pm (53.1 h)	β^−^ (100%)	285.9 (3.1)	1,071 (95.9)	Therapy
^157^Dy (8.1 h)	EC (100%)	326.2 (92)	-	Imaging
^165^Dy (2.3 h)	β^−^ (100%)	94.7 (3.6)	1,192 (15), 1,287 (83.0)	Therapy
^166^Dy (81.6 h)	β^−^ (100%)	82.5 (14)	404 (97.0)	Therapy
^166^Ho (26.8 h)	β^−^ (100%)	80.6 (6.7)	1,774 (49.9), 1,855 (48.8)	Therapy
^167^Tm (9.2 d)	EC (100%)	207.8 (42)	-	Imaging
^170^Tm (128.6 d)	β^−^ (99.86%), EC (0.13%)	84.2 (2.5)	968 (81.9)	Therapy
^172^Tm (63.6 h)	β^−^ (100%)	1093.6 (6.0), 1387.1 (5.6), 1529.7 (5.1)	414 (10.1), 1,801 (36), 1,880 (29)	Therapy
^169^Yb (32.0 d)	EC (100%)	109.8 (17.5), 130.5 (11.3), 177.2 (22.2), 197.9 (35.8), 307.7 (10.0)	-	Therapy
^177^Lu (6.7 d)	β^−^ (100%)	112.9 (6.4), 208.36 (11)	175 (11.7), 384 (8.9), 497 (79.4)	Therapy

* *https://www.nndc.bnl.gov/nudat2/ (21)*.

The research and interest on terbium radionuclides for human application has augmented many folds after the initiation of the CERN MEDICIS (Medical Isotopes Collected from ISOLDE) project. This new project aims to serve mankind by producing clinically important radionuclides to be supplied at the local hospitals. The project was conceptualized in 2012 (23) and on January 15, 2018, CERN announced that the CERN-MEDICIS facility had produced its first radioisotope, a batch of terbium (^155^Tb), part of the ^149, 152, 155, 161^Tb family (24).

These four radioisotopes of terbium can provide suitable matched pairs for theranostic activities. The first *in vivo* proof-of-concept in favor of the unique quadruplet family: ^149^Tb, ^152^Tb, ^155^Tb, ^161^Tb was reported by Müller et al. (25). Because of identical chemical properties, formulation of RPs with identical pharmacokinetics for these species are easily possible. The potential uses of these four terbium isotopes are given below. Table 2 provides at-a-glance use of these radioisotopes.

**Table 2:** Properties of four terbium radioisotopes [21, 25].

**Radio-isotope (*T*_**1/2**_)**	**Decay modes**	**Particle energy, *E*_**α**_ (MeV)**	**Particle energy, *E*_**β*avg***_ (MeV)**	***E*γ, keV; (*I*γ %)**	**Comment**
^149^Tb (4.12 h)	EC (82.3 %), α (17.7 %)	3.97 MeV; *I*_α_ = 16.7%	0.730 (Total *I*_β+_ = 7.1%)	165.0 (26.4)	Path length in normal tissue = 25–28 μm; LET = 140–142 keV/μm, use in α-therapy and/or PET (Annihilation 511 keV= 14.2%)
				352.2 (29.4)	
				388.6 (18.4)	
				652.1 (16.2)	
^152^Tb (17.5 h)	EC + β^+^ (100%)	_	1.140 (Total *I*_β+_ = 20.3%)	271.1 (9.5), 344.3 (63.5), 586.3 (9.2), 778.9 (5.5)	PET (Annihilation 511 keV= 41%)
^155^Tb (5.32 d)	EC (100 %)	_	_	105.3 (25.1), 180.1 (7.5), 262.3 (5.3)	SPECT
^161^Tb (6.89 d)	β^−^ (100 %)	_	0.154 (Total I_β−_= 101%)	25.6 (23.2), 48.9 (17.0), 74.6 (10.2)	β^−^ and/or auger therapy

### ^149^Tb

It is the only α-emitting radioisotope of Tb and became promising for targeted alpha therapy (TAT). With a tissue range of 25–28 μm and LET of 140–142 keV/μm, it can be conjugated with small-molecular weight carriers like peptides that are easily cleared from the body. ^149^Tb has additional features of emitting gamma rays (*E*_γ_ = 165 keV, *I*_γ_ = 26.4 %), which helps in its detection. At the same time, ^149^Tb is also β^+^ emitter.

**Figure 2:**
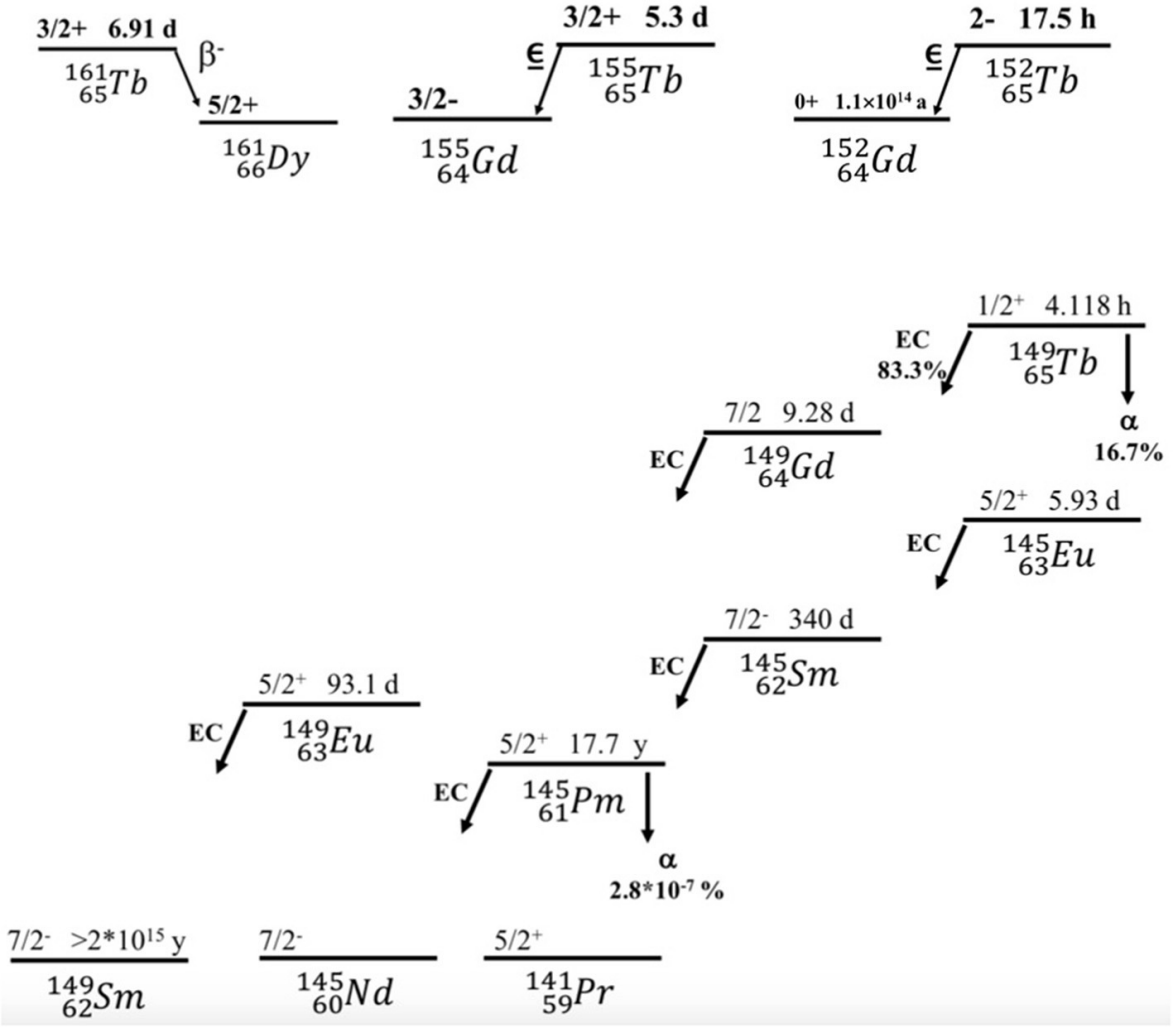
Decay series of Tb (^161^Tb, ^155^Tb, ^152^Tb, ^149^Tb) radioisotopes.

However, the major concern of ^149^Tb-TAT is its large-scale production. Another important concern about ^149^Tb-TAT is the decay scheme of ^149^Tb (Figure 2), which is quite complex. The daughter products of ^149^Tb are long-lived radionuclides, like ^149^Gd (9.28 d), ^145^Eu (5.93 d), ^145^Sm (340 d), ^149^Eu (93.1 d), etc. More research is required to elucidate any complexity arising due to *in vivo* presence of these ^149^Tb-decay products. For example, a preliminary dose evaluation related to retention of residual radioactivity after injection of 1 GBq ^149^Tb-rituximab conjugate in a patient's system was estimated at several time-intervals. It was estimated that after 1 year, 100 kBq ^149^Eu, 41 kBq ^145^Sm, 2.2 kBq ^145^Pm, and after 10 years, 50 Bq ^145^Sm, 3.1 Bq ^145^Pm will remain within the patient system (26). In such situations, bio-distribution profiling should be at par with ALARA principle. Several trials need to be carried out to reduce toxicity to non-target tissues.

### ^152^Tb

^152^Tb is a multiple β^+^- emitter with prominent end-point energies at 2,620 keV (5.9%) and 2,970 keV (8%). As a diagnostic tool, it is suitable for dosimetry and monitoring of ^149/161^Tb-radioligands. ^152^Tb can be the companion PET isotope in combination with other therapeutic radioisotopes in a theranostic approach. ^152^Tb is also a potential SPECT candidate due its multiple gamma lines. At the same time this multiple gamma-rays emission is a drawback when it is used as PET isotope due to increased radiation burden (27). To understand the bio-kinetic behavior of radio-lanthanides *in vivo*, Beyer (28) probed the efficacy of ^149^Tb and ^152^Tb in PET imaging, where it was realized that scan quality with ^152^Tb is significantly better than that obtained for ^149^Tb. Also, Beyer (28) indicated that ^152^Tb could be used for *in vivo* dosimetry to monitor ^149^Tb bio-distribution in radiotherapy.

### ^155^Tb

This radionuclide is a potential SPECT candidate. It can provide insight into the malignancy stages and may also be used for dosimetry calculation prior to therapy. In a matched-pair of ^155^Tb-^161^Tb, ^155^Tb may be beneficial for pre-therapeutic imaging and dosimetry prior to targeted therapy by ^161^Tb (29). With γ-energies at 87 keV (32%) and 105 keV (25%), ^155^Tb may have further applications in gamma camera scintigraphy (30). Recently, the clinical use of ^152^Tb- DOTATOC as human PET/CT agent was evaluated by Baum et al. (31).

### ^161^Tb

^161^Tb has interesting decay characteristics that make it a promising radionuclide in nuclear oncology. ^161^Tb mainly decays by release of β^−^ particles, but it also emits Auger electrons. It is believed that high LET of Auger electrons can be effective in reducing the survival capacity of cancer cells. On an average, 2.24 Auger and conversion electrons are emitted along with one beta-particle per decay (21, 30). Based on the pre-clinical studies and comparison with ^177^Lu, use of ^161^Tb for cancer therapy showed minimal or nil side effects to kidneys (29). According to theoretical simulations, in many cases ^161^Tb proves to be a better therapeutic candidate when compared to prevalent standard and non-standard therapeutic radioisotopes (32).

## Production of Terbium Radionuclides

The production in sufficient amounts and its separation in a no-carrier-added (NCA) state from the target matrix are the two most important criteria for any radionuclide to be used in the field of nuclear medicine. But in practice, a hurdle lies in the production of terbium radionuclides in an adequate quantity [except ^161^Tb, which can be produced in a reactor following a ^160^Gd(n, γ)^161^Gd(β^−^)^161^Tb reaction]. All possible production routes, i.e., (a) light ion induced reactions, (b) heavy ion induced reactions, and (c) spallation reactions have been exploited by scientists all over the world. Literature on their production and excitation function dates back to 1963. An overview of such attempts has been described below in nutshell.

### Production of Terbium Radionuclides by Light Charged Particle Activation

Large numbers of neutron deficient clinically important radionuclides are produced in particle accelerators by light charged particle activation. The production cross sections of these radionuclides by light charged particle induced reactions are usually very high, which is not exactly true for terbium radionuclides.

The radionuclide ^149^Tb can be produced by ^152^Gd(p,4n) reaction, which has several distinct disadvantages. The most important is that the natural abundance of ^152^Gd is only 0.2%, and at present about a 30% level of enrichment is possible. Due to partial enrichment, the reaction channels from other Gd isotopes would open up and the final product would be contaminated by other longer-lived terbium and rare earth isotopes. Moreover, the radiochemical separation of NCA ^149^Tb from neighboring bulk target or other co-produced radionuclides is a difficult task due to the similar chemical properties of the lanthanide elements.

Steyn et al. (33) had measured the cross sections of proton-induced reactions on ^152^Gd, ^155^Gd, and ^159^Tb with emphasis on the production of clinically important terbium radionuclides in a new generation commercially available 70 MeV cyclotron. The measured data was compared with different Monte Carlo simulation codes like ALICE. The authors have shown very high thick target yield of ^149^Tb and ^152^Tb is possible through the nuclear reactions of ^152^Gd(p,4n)^149^Tb and ^155^Gd(p,4n)^152^Tb, respectively, provided highly enriched targets of ^152^Gd and ^155^Gd are used. It should be noted that close to 100% enrichment level of ^155^Gd is possible. In the energy window of 66-30 MeV, production of 2,556 MBq/μAh of ^149^Tb and 1,924 MBq/μAh of ^152^Tb is achievable. However, due to the opening up of other reaction channels and also the impurity of other Gd isotopes in the target matrix, there will be Dy and Tb radionuclides contamination in both the ^149^Tb and ^152^Tb fractions. The indirect production route through ^159^Tb(p,5n)^155^Dy(ε)^155^Tb can also provide high yields of ^155^Tb. The advantage of this route is that ^159^Tb is the only naturally occurring stable isotope of terbium. The prominent disadvantage is that the product ^155^Tb is not in a no-carrier-added state, and always associated with bulk terbium. Also the yield would be contaminated by other dysprosium radioisotopes and their daughter products through ^159^Tb(p,xn)^153, 157, 159^Dy(ϵ)^153, 157, 159^Tb reactions. A highly efficient chemistry can exclude the dysprosium radionuclides but neither the bulk terbium nor the isotopic impurities of other terbium radionuclides.

On the contrary, Güray et al. (34) measured cross section of ^152^Gd(p,n)^152^Tb reaction in a much lower energy range. The astrophysical gamma process was the motivation behind their experiment. Nevertheless, they observed about ~101 mb cross section at 8 MeV for the above reaction. However, along with ^152^Tb, ^153^Tb would be co-produced *via* ^152^Gd(p,γ)^153^Tb reaction with ~4 mb cross-section at 8 MeV. Another interesting experiment with comparatively low energy proton for production of ^152, 155^Tb was carried out in Garching tandem accelerator (35). They produced ^152^Tb by irradiating a unique ion-implanted ^152^Gd target (enrichment > 99%) with 8 and 12 MeV protons. The main purpose of this experiment was to determine activity ratios of potential co-produced radionuclides with respect to ^152^Tb. They concluded that 12 MeV proton energy is suitable for ^152^Tb production with <1% contamination from ^153^Tb. The radioisotopic purity of ^152^Tb can further be improved by playing with the thickness of the target and reducing the proton beam energy to 10 or 11 MeV.

Recently, Formento-Cavaier et al. (36) measured the production cross section and yield of ^149^Tb from the irradiation of a natural gadolinium target with a 70-58 MeV proton beam. They also evaluated the production cross section of other co-produced terbium radionuclides. However, they recorded only 7.1 mb production cross section of ^149^Tb at 69.8 MeV projectile energy, produced through ^nat^Gd(p,x)^149^Tb reaction. At the same energy, the cross sections of other terbium radionuclides were measured as 31 mb (^150^Tb), 96 mb (^151^Tb), 114 mb (^152^Tb), and 124 mb (^153^Tb). Authors estimated that ~40 MBq/ μAh integrated yield of ^149^Tb in 2.74 mm thick Gd target could be possible. But the desired radioisotope would be contaminated by the comparatively longer-lived Tb radioisotopes.

Similarly, deuteron-induced reactions were also studied for terbium radionuclide production. Exhaustive theoretical and experimental cross section data of ^nat^Gd(d,xn)^151, 152, 153, 154, 155, 156, 160, 161^Tb reactions in the projectile energy range 5–50 MeV have been provided by Tárkányi et al. (37). Though considerably good cross section was obtained but the production of long-lived Tb isotopes could not be avoided along with the useful Tb radionuclides. Szelecsényi et al. (38) had re-measured excitation function for the ^nat^Gd(d,xn)^155^Tb and ^nat^Gd(d,xn)^161^Tb reactions from 4.2 to 21 MeV projectile energy. The cross-section of ^155^Tb is considerably high but the co-formation of long-lived Tb radioisotopes made the process unacceptable. Similarly, the amount of ^160^Tb would be higher than ^161^Tb, therefore it is also not possible to produce only ^161^Tb by deuteron irradiation. Authors decisively concluded that low energy deuteron irradiation either on a natural or on a highly enriched Gd target would not produce isotopically pure NCA ^155^Tb or ^161^Tb.

Duchemin et al. (39) measured the excitation function of ^nat^Gd(d,x)^151, 152, 153, 154m1, 154m2, 155, 156g, 160, 161^Tb over the deuteron energy range 10-34 MeV. Though the highest production cross section of ^155^Tb was observed at 24.6 MeV deuteron beam, again it would be contaminated by other Tb and Gd radioisotopes.

Zagryadskii et al. (40) examined the efficacy of the production of ^149^Tb through ^151^Eu(^3^He,5n)^149^Tb reaction in the energy range of 70-40 MeV. The thick target yield of ^149^Tb was 129 MBq/μAh, which is a considerably high yield for *in vivo* application. However, high radioisotopic impurities due to other exposed reaction channels were also observed. For example, ^3^He bombardment on ^151^Eu co-produced 75 MBq/μAh ^148^Tb, 335 MBq/μAh ^150^Tb, 845 MBq/μAh ^151^Tb, and 98 MBq/μAh ^152^Tb along with the desired ^149^Tb radionuclide. Therefore, though a high production rate is observed for ^149^Tb or ^152^Tb, unless the technology is developed to couple highly efficient isotope separation technique with commercial 70 MeV cyclotron, the high yield is practically of no use. Moiseeva et al. (41) also reported the same route, i.e., production of ^149^Tb by irradiation of 97% enriched ^151^Eu target with 70 MeV ^3^He. They calculated about 38.7 ± 7.7 MBq/μAh thick target yield of ^149^Tb through ^151^Eu(^3^He,5n) reaction (*E*_p_ = 70–30 MeV). The yield would be quite good for successful administration into patient's body but unfortunately the yields of ^150, 151, 152^Tb are much higher than ^149^Tb. Therefore, possibility for production of radioisotopically pure ^149^Tb is ruled out.

In the case of α-induced reactions, ^152^Gd(α,7n)^149^Dy(ε)^149^Tb reaction would give the highest yield, but the required projectile energy is of the order of 100 MeV, commonly unavailable in commercial cyclotrons. Moreover, the abundance of ^152^Gd remains too low (42).

Light particle induced reactions on some particular isotopes like ^152^Gd exhibits very high production cross sections of terbium radionuclides. However, natural abundance of these isotopes is very low, and sufficient technological advancement is required to increase the enrichment factors of these isotopes to a considerable level. Even if highly enriched targets are available, one cannot avoid production of other long-lived terbium radionuclides. To resolve this issue, commercial cyclotrons should be coupled with an isotope separator. Moreover, in some cases, as discussed above, higher projectile energy (e.g., 50 MeV proton or 100 MeV α) is required for high production yield of terbium radionuclides. Productions of terbium radionuclide in high quantity with high radioisotopic purity, especially production of ^149^Tb (the key radionuclide in terbium quadruplet) by light charged particle (p, d, ^3^He or ^4^He) induced reactions is not possible to date. Therefore, scientists have also explored the possibility of terbium quadruplet production by heavy ion activation.

### Production of Terbium Radionuclides by Heavy Ion (HI) Activation

Various nuclear reactions for heavy ion induced productions of terbium radioisotopes have been theoretically and experimentally explored for a long time. Amongst these ^141^Pr(^12^C,xn)^149−151^Tb, ^nat^Nd(^12^C,xn)^149, 150−153^Dy(ε)^149, 150−153^Tb, ^142^Nd(^10^B,3n)^149^Tb, ^142^Nd(^11^B,4n)^149^Tb, ^144^Nd(^10^B,5n)^149^Tb, ^140^Ce(^14^N,5n)^149^Tb, ^nat^Ce(^16^O,xn)^149, 151−153^Dy(ε)^149, 151−153^Tb, and ^139^La(^16^O,xn) ^149, 151, 152^Tb are noteworthy to mention.

Alexander and Simonoff (43) measured excitation functions of 12 heavy ion induced reactions that produce ^149^Tb. They used different projectiles like ^10^B, ^11^B, ^12^C, ^14^N, ^15^N, ^16^O, ^18^O, and ^19^F in combination with a variety of target isotopes from Ba to Nd, among which ^141^Pr is the only naturally abundant mononuclide target. Later, Kossakowski et al. (44) measured cross section of ^141^Pr(^12^C,4n)^149^Tb. Interestingly the cross section was two orders of magnitude higher than that of Alexander and Simonoff (43). This discrepancy and the importance of ^149^Tb prompted Maiti (45) to re-measure the excitation function of ^141^Pr(^12^C,4n)^149^Tb reaction over a 79–44 MeV incident projectile energy range. The results of Maiti (45) were not encouraging for heavy ion assisted production of ^149^Tb and supported the low cross section values earlier reported by Alexander and Simonoff (43). Beyer et al. (42, 46) also attempted the production of ^149^Tb *via* ^141^Pr(^12^C,4n)^149^Tb and ^142^Nd(^12^C,5n)^149^Dy(ε)^149^Tb reactions at JINR Dubna. In the case of the Nd target, Beyer et al. (42) achieved a reasonably higher yield, 2.2 MBq/μ*Ah* at the EOB. However, in this report the authors were silent about the co-produced radionuclides.

It is noteworthy to mention that many of the early attempts for heavy ion assisted production of terbium radionuclides were contributed from our group. For example, CeO_2_ target was irradiated with 80 MeV ^16^O, which produced ^151, 152, 153^Dy and their daughter products ^151, 152, 153^Tb in the matrix (47). We also calculated theoretical excitation function of ^140^Ce(^16^O,4n)^152^Dy, ^142^Ce(^16^O,6n)^152^Dy along with ^140, 142^Ce(^16^O,xn)^151, 153^Dy by Monte Carlo simulation code PACE 2. Interestingly the production cross sections of ^151, 153^Dy were found to be much higher than ^152^Dy. Studies on the production of terbium radionuclides were further continued by irradiating an Nd_2_O_3_ target with ^12^C, which produced ^150−153^Dy and their daughter products ^150−153^Tb radionuclides in the matrix (48). However, the yield of the radionuclides was low and not sufficient for *in vivo* applications. Due to the restrictions of BARC-TIFR pelletron (Mumbai, India), we could irradiate the Nd_2_O_3_ target with a maximum 83 MeV ^12^C beam. Here also, excitation functions of ^142, 144, 146^Nd(^12^C,xn)^150, 151, 152, 153^Dy reactions were calculated by PACE 2 code. It was found that the production cross section of ^152^Dy is comparable with those of ^150, 151, 153^Dy and therefore ^152^Tb (decay product of ^152^Dy) would always be contaminated by longer-lived isotopes of Dy and Tb. We have also attempted production of ^151, 152^Tb by ^16^O irradiation on La_2_O_3_ target (49). The advantage of this method is that terbium radionuclides are directly produced through ^139^La(^16^O,xn)^151, 152^Tb reaction, not through the decay of dysprosium radionuclides like earlier examples.

In Table 3 we have provided a concise picture of various attempts of terbium radionuclides production by light and heavy ion induced reactions.

**Table 3:** Available production cross section data of terbium radionuclides.

**Nuclear reaction**	**Projectile energy, MeV**	**Cross section (σ_max_), mb**	**Comment**	**References**
^152^Gd(p,4n)^149^Tb	41.31	248		(33)
^155^Gd(p,4n)^152^Tb	48.2	821		
^nat^Gd (p,x)^149^Tb	69.8	7	^150, 151, 153^Tb will also be produced in considerable amount	(36)
^nat^Gd (p,x)^152^Tb		114		
^152^Gd(p,n)^152^Tb	8	101	^153^Tb is co-produced with 4 mb cross section	(34)
^159^Tb(p,n)^152^Tb	97	244		(50)
^nat^Gd (d,x)^152, 155, 161^Tb	49.2	^152^Tb = 98.2	^151, 154, 156, 160^Tb isotopes are co-produced	(37)
	42.1	^155^Tb = 376		
	10.9	^161^Tb = 234		
^nat^Gd (d,x)^152, 161^Tb	21.1	^155^Tb = 269	^156, 160^Tb isotopes are co-produced	(38)
	9.6	^161^Tb = 39		
^nat^Gd (d,x)^152, 155^Tb	33.34	^152^Tb = 14.4	^153, 154m1, 154m2, 156g, 160^Tb and ^153, 159^Gd isotopes are co-produced in high quantity	(39)
	24.56	^155^Tb = 317.7		
^nat^Gd (a,x)^155^Tb	73.4	304.43		(51)
^141^Pr(^12^C,xn)^149^Tb	77.4	408		(44)
^141^Pr(^14^N,p5n)^149^Tb	101	220		
^141^Pr(^12^C,xn)^149−151^Tb	62.1	^149^Tb = 27.3	Very high production cross section of ^149^Gd	(45)
	52.9	^150^Tb = 36.4		
	54.1	^151^Tb = 32.7		
^152^Sm(^7^Li,4n)^155^Tb	38	669		(52)

### Production of Terbium Radionuclides by Spallation Reaction

The other option left for the production of terbium radionuclides with high purity and in high quantity is spallation induced reactions. One of the major constraints of spallation reaction is that such high energy facilities are limited to only few centers worldwide. Nevertheless, a glimpse of vibrant research carried out in such advanced centers to produce terbium radionuclides has been given below.

Literature is available on the production of ^149^Tb by spallation reaction even in 1966, though the aim of the experiment was something else. Franz and Friedlander (53) measured the production cross section of ^149^Tb from 0.6 to 30 GeV proton induced reaction on Au target. The reported cross sections were rather low, e.g., for 1.4 GeV proton beam, the production cross section of ^148^Tb was only about 10 mb. Heydegger and Van Ginneken (54) re-measured the production cross section of ^149^Tb produced from 0.2 to 0.4 GeV proton induced reaction on gold target. They reported a still lower cross section, ~5 μb at 0.4 GeV energy.

The CERN-ISOLDE has the lead role in research on production of terbium quadruplet radioisotopes by the impact of high energy proton. Allen et al. (55) irradiated a Ta foil target by 1 GeV protons at CERN proton accelerator in order to produce radio-lanthanides by spallation reaction. The products of *A* = 152 were collected by an online mass separator at the ISOLDE on high purity Al catcher foil, and later on, they detected ^152^Tb in the catcher foil in considerable amounts. Beyer et al. (42) irradiated a thick Ta foil (112 g cm^−2^) by 1–2 μA integrated beam current, 1.4 GeV proton beam from the CERN PS booster and obtained about 500 MBq ^149^Tb at the end of collection (EOC), after 4–8 h bombardment. Later on, isobars of 149 mass numbers were collected using the ISOLDE facility at CERN into a thin layer of KNO_3_, which was molten on Al-backings. In another experiment (31), a Ta-foil implanted into thin KNO_3_ layer on an aluminum holder was irradiated with 1.4 GeV protons at the CERN-ISOLDE facility. In both the experiments, radiochemical separation of the desired isotope was required, which has been described in section Chemical Separation of Terbium Radionuclides From the Target Matrix.

Recently, Verhoeven et al. (56) measured the spallation cross sections for the production of ^149^Tb from a tantalum target at different proton energies from 0.3 to 1.7 GeV. They observed that the highest production cross section of ^149^Tb from a Ta target is around 1.1 and 1.3 GeV energy range. From this data they concluded that the operating energy at CERN-ISOLDE (1.4 GeV) is not optimum for ^149^Tb production. A lower proton energy (1.3 GeV) would give a much higher yield of ^149^Tb.

In the CERN-MEDICIS facility, the terbium radionuclides, ^149^Tb, ^152^Tb, and ^155^Tb are produced in 1.4 GeV proton induced spallation on Ta-Re targets placed behind the ISOLDE-HRS target. Typical irradiation lasts for 12–16 h. About 38 GBq ^149^Tb, 37 GBq ^152^Tb, and 5.3 GBq ^155^Tb in target activity can be produced. However, the extraction efficiency is ~1%, with a wide scope for improvement. The ^161^Tb is produced in UC_x_-Re target with in-target activity of 19 MBq and 1% extraction efficiency.

^161^Tb can also be produced after bombardment of neutrons on highly enriched ^160^Gd targets at spallation neutron source (SINQ) of Paul Scherrer Institute (PSI), Switzerland or at a high-flux nuclear reactor at Laue-Langevin (ILL) situated in France (57). The SPES-ISOLPHARM facility in Italy is also planning to produce ^152^Tb, ^155^Tb, ^156^Tb, ^161^Tb radioisotopes by high flux protons bombardment on Gd targets (58).

In a recent experiment, our group had irradiated lead bismuth eutectic (LBE) targets at CERN-ISOLDE by a 1.4 GeV proton beam. The LBE targets have been proposed as converter targets and would be used worldwide in next-generation RIB facilities. We have assessed all the radionuclides produced by the interaction of 1.4 GeV proton beam in the LBE matrix and found numbers of clinically important radionuclides including some of the radionuclides of the Swiss-knife family. It is estimated that about 4.5 MBq/μ*Ah* ^149^Tb and ^151^Tb will be produced in 50 mm long and 6 mm diameter LBE targets, which is a considerable amount for *in vivo* applications if it can be separated using ISOL or similar facilities (59).

## Chemical Separation of Terbium Radionuclides From the Target Matrix

For *in vivo* application, any radionuclide should be free from the target matrix and chemistry has an important role. There are only a few reports available in literature related to the radiochemical separations of terbium radionuclides. Earlier, production of NCA ^161^Tb was attempted by Subhodaya et al. (60) by the neutron activation of natural gadolinium, and subsequent β decay of ^161^Gd to ^161^Tb. The NCA ^161^Tb was separated from gadolinium target by Dowex 50 resin using α-hydroxybutyric acid (HIBA) at pH 4.4 as eluent. All the lanthanide elements have similar properties. The separation is even difficult and a colossal task when no-carrier-added lanthanide has to be separated from the adjacent bulk amount lanthanide. Though Subhodaya et al. (60) reported considerable amounts of separation of ^161^Tb from the bulk gadolinium target, they could not achieve enough purity required for clinical application.

Liquid Liquid extraction (LLX) technique was utilized to separate ^151, 152, 153^Dy, ^151, 152, 153^Tb from the ^16^O irradiated ceric oxide target (47). The irradiated target was dissolved in a mixture of conc. HNO_3_ and H_2_SO_4_ acid, evaporated to dryness, and finally taken into 10^−3^ M HCl medium. The liquid cation exchanger, di-(2-ethylhexyl)phosphoric acid (HDEHP) dissolved in cyclohexane was used as extractant. An excellent separation was achieved where the organic phase contained only ^151, 152, 153^Tb (~90% chemical yield) without any contamination of co-produced Dy radionuclides or bulk Ce target. Similarly, an attempt was made to separate NCA ^150−153^Tb radionuclides from ^12^C irradiated Nd_2_O_3_ target and the co-produced ^150−153^Dy radionuclides (48). The same reagent, i.e., HDEHP was used and ^150−153^Tb could be separated with high radiochemical purity.

As discussed in section Production of terbium radionuclides by spallation reaction, Beyer et al. (42) bombarded a Ta target with a 1.4 GeV proton beam for 4–8 h and collected the *A* = 149 isobars. Since the half-life of ^149^Tb is comparable to the time of collection, the ^149^Tb was contaminated by its decay products, ^149^Gd and ^149^Eu. Moreover, ^133^Ce and ^133^La in the form of pseudo-isobaric ions, ^133^CeO^+^ and ^133^LaO^+^, also contaminated the ^149^Tb fraction. Therefore, radiochemical separation was mandatory to get pure ^149^Tb. Beyer et al. (42) separated the radio-lanthanides by cation exchange chromatography with Aminex A5 resin. The radio-lanthanides were eluted with α-hydroxyisobutyric acid (α-HIBA) at pH 5.0. ^149^Tb was eluted first followed by ^149^Gd and ^149^Eu. The pseudo-isobars ^133^CeO^+^ and ^133^LaO^+^ were eluted at the end. A similar study on the chemical separation of ^155^Tb from pseudo-isobaric ^139^Ce^16^O was reported by Webster et al. (61). In this study, sodium bromate was used to oxidize Ce(II) to Ce(IV). Pre-packed commercial resins like UTEVA, TEVA, TK100, and AG1 were used for extraction chromatographic studies. 8 M HNO_3_ could elute ^155^Tb without contamination from Ce, which was later eluted by 0.1 M HCl.

Aziz and Artha (62) reported the separation of ^161^Tb from bulk Gd target by extraction chromatography using LN resin. ^161^Tb was produced by thermal neutron irradiation of natural Gd_2_O_3_. The bulk Gd was eluted first by 0.8 M HNO_3_ followed by elution of ^161^Tb by 3 M HNO_3_. Authors reported that about 70% of ^161^Tb could be recovered with >99% radionuclide purity.

Maiti et al. (63) irradiated natural praseodymium target with 72 MeV ^12^C beam and produced NCA ^149, 150, 151^Tb radionuclides along with ^149^Gd in the matrix. After production, the NCA terbium radionuclides were separated from the target by LLX using HDEHP/cyclohexane as liquid cation exchanger. The terbium radionuclides, ^151, 152^Tb were extracted in the organic phase and was back extracted by DTPA. As high as a 10^5^ separation factor was achieved between bulk Pr and NCA Tb radionuclides (63).

Therefore, the role of chemical separation cannot be ignored even in the presence of the ISOL technique. In Table 4 we have provided the list of radioanalytical chemistry developed so far for separation of terbium radionuclides.

**Table 4:** Radiochemical separation of no-carrier-added terbium radionuclides.

**Nuclear reaction**	**Radiochemical separation**	**Separation factors (*S*)**	**References**
Gd(n,γ)^161^Gd(ε)^161^Tb^.^	Extraction using DOWEX 50 resin, and α- HIBA as. eluent at pH 4.4		(60)
^nat^Nd(^12^C,xn)^150−153^Dy(ε)^150−153^Tb	LLX: HDEHP/cyclohexane and HCl	*S*_Tb/Nd_ = 100	(48)
		*S*_Dy/Nd_ = 390	
		*S*_Tb/Dy_ =38	
^nat^Ce(^16^O,xn)^151−153^Dy(ε)^151−153^Tb	LLX: HDEHP/cyclohexane and HCl	*S*_Tb/Ce_ = 657	(47)
		*S*_Dy/Ce_ = 41,157	
		*S*_Tb/Dy_ = 38	
^141^Pr(^12^C,xn)^149−151^Tb	LLX: HDEHP/cyclohexane and HCl	*S*_Tb/Pr_ = 470,000	(63)
		*S*_Gd/Pr_ = 394	
		*S*_Tb/Gd_ = 52,000	
^139^La(^16^O, xn) ^149, 151, 152^Tb	LLX: HDEHP/cyclohexane and HCl	*S*_Tb/La_ = 816	(49)
Proton induced spallation on Ta target, followed by collection of A = 149 fraction by ISOL which contained ^149^Tb, ^149^Gd, ^149^Eu, ^133^CeO^+^, ^133^LaO^+^	Adsorption in cation exchange; AMINEX-A5, followed by elution with α-HIBA	^149^Tb was eluted first without contamination from other radionuclides	(42)
^142^Nd(^12^C,5n)^149^Dy(ε)^149^Tb	Tb, Gd separation, elution with α-HIBA		(64, 65)
Separation study of isobaric ^149^Tb and ^133^Ce	Extraction chromatography using UTEVA, TEVA, TK-100, AG-1 resin, 8 M HNO_3_	^149^Tb was eluted first, later Ce was eluted by HCl	(61)
Gd(n,γ)^161^Gd(ε)^161^Tb	Extraction chromatography using LN resin, 0.8 M and 3 M HNO_3_		(62, 66)

## Conclusion

The use of terbium radionuclides for TAT, to deliver very small radiation doses exactly where they are needed to avoid destroying the surrounding healthy tissues, would be a great jump in the field of nuclear medicine. However, the research with the theranostics terbium quadruplet radionuclides are limited mainly in and around Geneva city. Only a handful numbers of pre-clinical trials have been conducted. Many more such studies are required before their direct administration to the human body for therapy or diagnosis. The main constraint is the limited scope for production of the terbium radionuclides in sufficient amounts due to the costs of highly enriched targets, low reaction cross section, radioactive impurities, presence of non-radioactive isotope, etc. Isotope separation on-line (ISOL) has become the much-sorted accelerator technology at present and is also the future to solve the riddle of terbium isotope production. Following the CERN's success, many mega facilities for RIB research, like ISAC, Canada; ISOL@MYRRHA, Belgium; J-PARC ISOL, Japan; ISOLPHARM, Italy, etc., have now dedicated some of their programs to medical research and the production of isotopes. In fact, dedication of research toward the direct benefit of mankind will also help these centers to sustain the research on fundamental science. However, one has to keep in mind that the research facilities can only help to start medical programs to make the proof of concept. It is important that the industry should take over at some points to consolidate production. It is also necessary to develop techniques that are affordable and easy to handle.

## Author Contributions

NN and SL: conceptualization, primary literature search, writing of the drafts, and corrections. All authors contributed to the article and approved the submitted version.

## Conflict of Interest

The authors declare that the research was conducted in the absence of any commercial or financial relationships that could be construed as a potential conflict of interest.

## Abbreviations

ALARA: As Low As Reasonably Achievable (taking economic and social factors into account)
DNA: Deoxyribonucleic Acid
DOTA: Dodecane Tetraacetic Acid (1,4,7,10-tetraazacyclododecane-1,4,7,10-tetraacetic acid)
DTPA: diethylenetriamine pentaacetic acid
EC: Electron Capture
EOB: End of Bombardment
EOC: End of Collection
HDEHP: di-(2-ethylhexyl) phosphoric acid
HI: Heavy Ion
HIBA: α-hydroxy butyric acid
ISOL: Isotope Separation Online
LBE: Lead Bismuth Eutectic
LET: Linear Energy Transfer
LLX: Liquid Liquid Extraction
Mab: Monoclonal Antibody
NCA: No-Carrier-added
NET: Neuro Endocrine Tumor
NOTA: Nonane Tetraacetic Acid (1,4,7-triazacyclononane-1,4,7-triacetic acid)
PET: Positron Emission Tomography
PRRT: Peptide Receptor Radionuclide Therapy
RBE: Relative Biological Effectiveness
RIB: Radioactive Ion Beam
RP: Radiopharmaceutical
SPECT: Single-Photon Emission Computed Tomography
TAT: Targeted Alpha Therapy.
